# Seven-Spot Ladybird Optimization: A Novel and Efficient Metaheuristic Algorithm for Numerical Optimization

**DOI:** 10.1155/2013/378515

**Published:** 2013-12-09

**Authors:** Peng Wang, Zhouquan Zhu, Shuai Huang

**Affiliations:** School of Marine Science and Technology, Northwestern Polytechnical University, Xi'an 710072, China

## Abstract

This paper presents a novel biologically inspired metaheuristic algorithm called seven-spot ladybird optimization (SLO). The SLO is inspired by recent discoveries on the foraging behavior of a seven-spot ladybird. In this paper, the performance of the SLO is compared with that of the genetic algorithm, particle swarm optimization, and artificial bee colony algorithms by using five numerical benchmark functions with multimodality. The results show that SLO has the ability to find the best solution with a comparatively small population size and is suitable for solving optimization problems with lower dimensions.

## 1. Introduction

In recent years, heuristic algorithms have gained popularity because of their ability to find near-optimal solutions to problems unsolved by analytical methods within reasonable computation time due to the multimodality or high dimensionality of their objective functions [1]. Heuristic algorithms are usually developed to solve a specific problem. There is also a class of heuristic algorithms which can be used to solve a large class of problems either directly or with minor modifications, hence the name meta-heuristic algorithms [2].

Researchers continue to develop many meta-heuristic algorithms. Some of the most successful meta-heuristic algorithms include genetic algorithm (GA) [3], ant colony optimization [4], particle swarm optimization (PSO) [5], and artificial bee colony (ABC) [6]. Some of the recently proposed meta-heuristic algorithms include cuckoo search [7], monkey search [8], firefly algorithm [9], grenade explosion method [10], cat swarm optimization [11], and the artificial chemical reaction optimization algorithm [12]. The majority of these algorithms are biologically inspired; that is, they mimic nature for problem solving.

Meta-heuristic algorithms are widely used in different fields and problems, such as manufacturing [13], services [14], scheduling [15], transportation [16], health [17], sports [18], geology [19], and astronomy [20]. A single meta-heuristic algorithm that can solve all optimization problems of different types and structures does not exist, and, thus, new meta-heuristic optimization algorithms are continuously developed [21].

This paper introduces a novel biologically inspired meta-heuristic algorithm called seven-spot ladybird optimization (SLO). SLO is inspired by the foraging behavior of a seven-spot ladybird. This paper presents the basic concepts and main steps of the SLO and demonstrates its efficiency. The performance of the SLO is compared with some popular meta-heuristic algorithms, such as GA, PSO, and ABC, by using five different dimensional classical benchmark functions, as given in [6, 22]. The simulated results show that the SLO has the ability to get out of a local minimum and is efficient for some multivariable, multimodal function optimizations.

In general, all the metaheuristic algorithms have something in common in the sense that they are population-based search methods. These methods move from a set of points (population) to another set of points in a single iteration with likely improvement using a combination of deterministic or probabilistic rules. The most remarkable difference of these metaheuristic algorithms lies in the updating rules. The GA is inspired by the principles of genetics and evolution and mimics the reproduction behavior observed in biological populations. The GA employs the principal of “survival of the fittest” in its search process to select and generate individuals that are adapted to their environment. In PSO, instead of using genetic operators, each particle adjusts its “flying” according to its own flying experience and its companions' flying experience [23]. ABC uses minimal model that mimics the foraging behavior of bees comprising of employed bees, onlooker bees and scouts [24]. The bees aim at discovering places of food sources with high amount of nectar (good fitness values). Differently, in our paper, SLO attempts to simulate the foraging behavior of a seven-spot ladybird which is rarely researched in the field of metaheuristic algorithm. The SLO algorithm consists of three essential components: dividing patches, searching food, and dispersal. Dividing patches increases the search efficiency and dispersal progressively reduces the search space. The search strategy in our algorithm is classified into extensive search and intensive search. Extensive search overcomes the weakness of local search and intensive search increases the possibility of achieving latent best solution. All the ideas are inspired by recent discoveries on the foraging behavior of a seven-spot ladybird which are quite different from other metaheuristic algorithms.

The rest of this paper is organized as follows.  Section 2 presents the foraging behavior of the seven-spot ladybird.  Section 3 describes the SLO and the steps in detail.  Section 4 discusses the experiments and the results.  Section 5 draws the conclusions.

## 2. Seven-Spot Ladybird Foraging Behaviors

**Figure 1:**
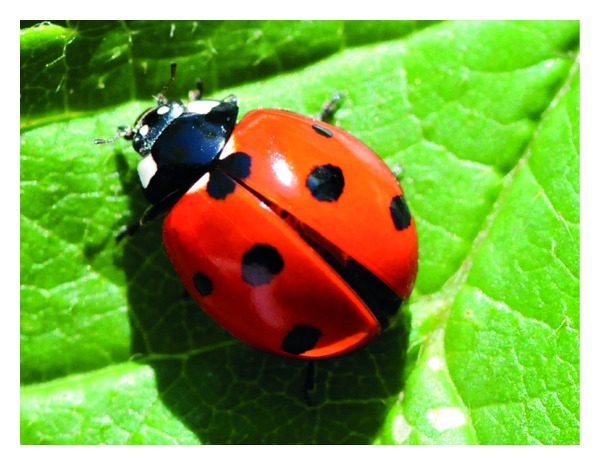
Seven-spot ladybird.

The seven-spot ladybird (Figure 1), *Coccinella septempunctata*, is a common, easily recognizable insect that has attracted a considerable amount of interest from professional entomologists.

**Figure 2:**
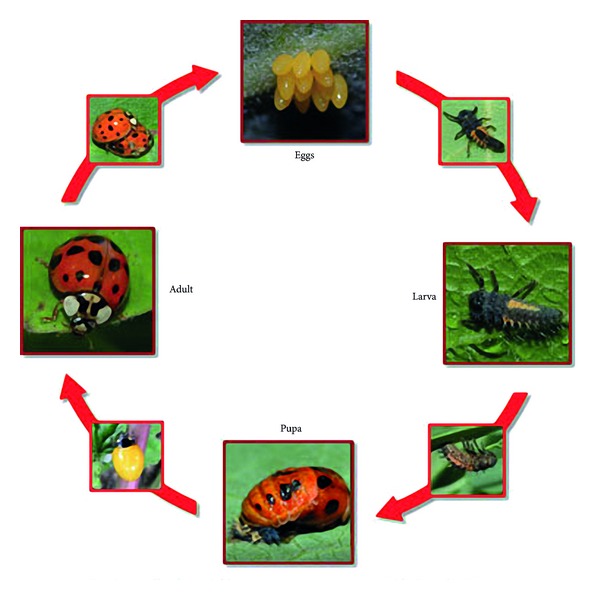
The life history of the seven-spot ladybird.

Recent studies have shown that seven-spot ladybirds are more social than we believe them to be [25–28]. Seven-spot ladybirds use different kinds of *pheromones* at different stages of its life history, such as eggs, larvae, pupa, and adult stages (Figure 2). Some chemical ecologies of the seven-spot ladybirds, with special attention to semiochemicals involved in *social communication* and *foraging behaviors*, have been reviewed in [29].

**Figure 3:**
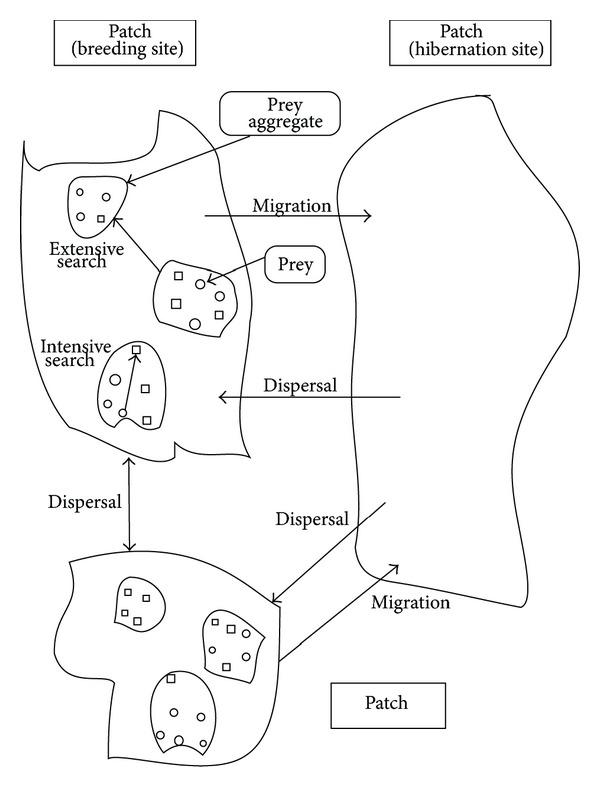
Diagram illustrating how a ladybird might perceive its environment and forage for resources.

Seven-spot ladybirds are effective predators of aphids and other homopteran pests, and, thus, their foraging behaviors have been extensively studied [30–35]. Some scholars classified the *environmental levels* of seven-spot ladybirds into prey, patches, and habitats (Figure 3) [33–35], providing a framework for discussing the foraging behaviors of seven-spot ladybirds.

In Figure 3, movement between prey within aggregates of aphids is referred to as *intensive search* which is slow and sinuous. Movement between aggregates within a patch is referred to as *extensive search* which is relatively linear and fast. Movement between patches is called *dispersal* and movement from patches to hibernation is called *migration*.

Seven-spot ladybirds locate their prey via extensive search and then switch to intensive search after feeding. While searching for its prey, a seven-spot ladybird holds its antennae parallel to its searching substratum and its maxillary palpi perpendicular to the substratum. The ladybird vibrates its maxillary palpi and turns its head from side to side. The *sideward vibration* can increase the area wherein the prey may be located.

How seven-spot ladybirds decide when to leave a patch for another, also known as *dispersal*, remains unclear. Several authors suggested that beetles decide to leave when the capture rate falls below a critical value or when the time since the last aphid was captured exceeds a certain threshold [36–38].

## 3. Proposed Seven-Spot Ladybird Optimization (SLO) Algorithm

This section describes the proposed seven-spot ladybird optimization (SLO) algorithm, which simulates the foraging behavior of seven-spot ladybirds to solve multidimensional and multimodal optimization problems. The main steps of the SLO are as follows.

**Step 1 (dividing patches)**

Suppose that the search space (environment) is a *D*-dimensional space. The *i*th dimensional space is divided into *n*_*i*_ subspaces, and the whole dimensional space is divided into *n* = Π*n*_*i*_ subspaces (patches).

**Step 2 (initializing population)**

Suppose that each seven-spot ladybird is treated as a point in a *D*-dimensional patch. The *i*th ladybird is represented as *X*_*i*_ = (*x*_*i*1_, *x*_*i*2_,…, *x*_*iD*_), where *X*_*i*_ is a latent solution to the optimized question.

If *m* is the number of seven-spot ladybirds initialized with random positions in a patch, then the population size of the seven-spot ladybirds is *N*, *N* = *m* × *n*.

**Step 3 (calculating fitness)**

For each particle, evaluate the optimization fitness in a *D*-dimensional patch.

**Step 4 (choosing the best ladybird)**

The current fitness evaluation of each ladybird was compared with the fitness value of its best historical position (*s*best). If the current value is better than the previous one, then set *s*best value is equal to the current value, and the *s*best position is equal to the current position.

The current best fitness evaluation of all the ladybirds in a patch was compared with the fitness value of their previous best position (*l*best). If current value is better than the previous one, then set *l*best value equal to the current value, and the *l*best position equal to the current position.

The current best fitness evaluation of all the ladybirds in the population was compared with the fitness value of their previous best position (*g*best). If the current value is better than the previous one, then set *g*best value equal to the current value, and the *g*best position equal to the current position.

**Step 5 (dispersal)**

In the SLO, if a position does not improve in a predetermined number of cycles, then a new position is produced in the patch where *g*best exists, replacing the abandoned position. The new position is produced near the *g*best to share the information of the best ladybird in the whole particle. The value of the predetermined number of cycles (*limit*) is an important control parameter in the SLO.

If the abandoned position is *X*_*i*_ and *j* ∈ {1,2,…, *D*}, then the seven-spot ladybird discovers a new position *X*_*i*_′ as follows:

(1) $$\begin{array}{c} x_{i,j}^{\prime }=x_{g\text{best},j}+\phi w, \end{array}$$

where *w* is the neighborhood space of *g*best and *ϕ* is a random number between [−1,1].

**Step 6 (updating positions)**

The position of a ladybird is updated associated with its previous movement. If a ladybird has done extensive search, then the position of the ladybird is changed as follows:

(2) $$\begin{array}{c} V_{i}\left(t\right)=c*r_{1}*\left(S_{i}\left(t\right)-X_{i}\left(t\right)\right)+\varepsilon_{1}, \end{array}$$

(3) $$\begin{array}{c} X_{i}\left(t+1\right)=X_{i}\left(t\right)+V_{i}\left(t\right),\;\;\left|V_{i}\left(t\right)\right|\leq V_{\max}. \end{array}$$

After intensive search, a ladybird switches to extensive search. The position is updated according to the following equations:

(4) $$\begin{array}{c} V_{i}\left(t\right)=c*r_{2}*\left(L_{i}\left(t\right)-X_{i}\left(t\right)\right)+\varepsilon_{2}, \end{array}$$

(5) $$\begin{array}{c} X_{i}\left(t+1\right)=X_{i}\left(t\right)+V_{i}\left(t\right),\;\;\left|V_{i}\left(t\right)\right|\leq V_{\max}. \end{array}$$

In (2) and (4), *r*_1_ and *r*_2_ are two random numbers uniformly distributed from 0 to 1 and the positive constant *c* is used for adjusting the search step and search direction in each iteration. In (3) and (5), the velocities of the ladybirds in each dimension are limited to the maximum velocity *V*_max⁡_, which decides the search precision of the ladybirds in a solution space. If *V*_max⁡_ is too high, then the ladybirds will possibly fly over the optimal solution. However, if the *V*_max⁡_ is too low, then the ladybirds will fall into the local search space and have no method to carry on with the global search. Typically, *V*_max⁡_ is set as follows:

(6) $$\begin{array}{c} V_{\max}=0.2\left(\text{ub}-\text{lb}\right), \end{array}$$

where ub and lb are the upper and lower bounds of each patch, respectively. Equation (6) came from [39]. We adopt it here to clamp the particles' velocities on each dimension.

From equations above, we can see that the velocity updating rule is composed of three parts. The first part, known as *intensive search*, is inspired by the slow and sinuous movements of ladybirds. The second part, known as *extensive search*, is derived from the relatively linear and fast movement behavior of ladybirds. The third part imitates the *sideward vibration* of ladybirds to increase the search area where the potential solution may exist. The parameter *ε*_1_ and *ε*_2_ are usually set as relatively small random numbers.

**Step 7 (inspecting termination condition)**

If the termination condition is satisfied, that is, the SLO has achieved the maximum iteration number, then the SLO is terminated; otherwise, it returns to Step 3.

## 4. Experiments

### 4.1. Benchmark Functions

In the field of heuristic computation, it is common to compare different algorithms using a set of test functions. However, the effectiveness of an algorithm against another algorithm cannot be measured by the number of problems that it solves better [40]. In this way, we have made a previous study of the functions to be optimized for constructing a test set with fewer functions and a better selection. We used five classical benchmark functions to compare the performance of the proposed SLO with those of GA, PSO, and ABC. This set is adequate to include different kinds of problems such as unimodal, multimodal, regular, irregular, separable, nonseparable, and multidimensional. Mathematical descriptions of the benchmark functions were obtained from [6, 22].

The first function is the Griewank function whose value is 0 at its global minimum (0,0,…, 0) (7). Initialization range for the function is [−600,600]. The Griewank function has a product term that introduces interdependence among its variables. The aim is to overcome the failure of the techniques that optimize each variable independently. The optima of the Griewank function are regularly distributed. Since the number of local optima increases with the dimensionality, this function is strongly multimodal. The multimodality disappears for sufficiently high dimensionalities (*n* > 30) and makes the problem unimodal. Consider

(7) $$\begin{array}{c} f_{1}\left(\vec{x}\right)=\frac{1}{4000}\left(\overset{D}{\underset{t=1}{\sum }}\left(x_{i}^{2}\right)\right)-\left(\overset{D}{\underset{t=1}{\prod }}\cos\left(\frac{x_{i}}{\sqrt{i}}\right)\right)+1. \end{array}$$

The second function is the Rastrigin function whose value is 0 at its global minimum (0,0,…, 0) (8). Initialization range for the function is [−15,15]. The Rastrigin function is based on the Sphere function with the addition of cosine modulation to produce many local minima, making it multimodal. The locations of the minima are regularly distributed. The difficult part about finding optimal solutions to the Rastrigin function is that an optimization algorithm is easily trapped in a local optimum on its way towards the global optimum. Consider

(8) $$\begin{array}{c} f_{2}\left(\vec{x}\right)=\overset{D}{\underset{t=1}{\sum }}\left(x_{i}^{2}-10\cos\left(2\pi x_{i}\right)+10\right). \end{array}$$

The third function is the Rosenbrock function whose value is 0 at its global minimum (1,1,…, 1) (9). Initialization range for the function is [−15,15]. The global optimum is inside a long, narrow, parabolic-shaped flat valley. Since it is difficult to converge to the global optimum, the variables are strongly dependent, and the gradients generally do not point towards the optimum, this problem is repeatedly used to test the performance of the optimization algorithms. Consider

(9) $$\begin{array}{c} f_{3}\left(\vec{x}\right)=\overset{D}{\underset{t=1}{\sum }}100{\left(x_{i}^{2}-x_{i+1}\right)}^{2}+{\left(1-x_{i}\right)}^{2}. \end{array}$$

The fourth function is the Ackley function whose value is 0 at its global minimum (0,0,…, 0) (10). Initialization range for the function is [−32.768,32.768]. The Ackley function has an exponential term that covers its surface with numerous local minima, making its complexity moderated. An algorithm that only uses the gradient steepest descent will be trapped in the local optima, but any search strategy that analyzes a wider region will be able to cross the valley among the optima and achieve better results. A search strategy must combine the exploratory and exploitative components efficiently to obtain good results for the Ackley function. Consider

(10) f4(x→)=20+e−20e(−0.2(1/D)∑t=1Dxi2)−e(1/D)∑t=1Dcos⁡⁡(2πxi).

The fifth function is the Schwefel function whose value is 0 at its global minimum (420.9867,420.9867,…, 420.9867) (11). Initialization range for the function is [−500,500]. The surface of the Schwefel function is composed of a large number of peaks and valleys. The Schwefel function has a second best minimum far from the global minimum where many search algorithms are trapped. Moreover, the global minimum is near the bounds of the domain. Consider

(11) $$\begin{array}{c} f_{5}\left(\vec{x}\right)=D*418.9829+\overset{D}{\underset{t=1}{\sum }}-x_{i}\sin\left(\sqrt{\left|x_{i}\right|}\right). \end{array}$$

### 4.2. Settings for Algorithms

The common control parameters for the algorithms include population size and number of maximum generation. In the experiments, maximum generations were 750, 1000, and 1500 for Dimensions 5, 10, and 30, respectively, and the population size was 50. Other control parameters of the algorithms and the schemes used in [6], including the control parameter values employed for GA, PSO, and ABC are presented below.

#### 4.2.1. GA Settings

The settings for the used GA scheme presented in [6] are as follows: single point uniform crossover with rate of 0.95, random selection mechanism, Gaussian mutation with rate of 0.1, and linear ranking fitness function. A child chromosome is added to the population by using the child production scheme.

#### 4.2.2. PSO Settings

PSO equations can be expressed as follows:

(12) v→(t+1)=wv→(t)+ϕ1rand(0,1)(p→(t)−x→(t))+ϕ2rand(0,1)(g→(t)−x→(t)),x→(t+1)=x→(t)+v→(t+1),

where *w* is the additional inertia weight that varies from 0.9 to 0.4 linearly with the iterations. The learning factors, *ϕ*_1_ and *ϕ*_2_, are set to 2. The upper and lower bounds for *v*, (*v*_min⁡_, *v*_max⁡_) are set as the maximum upper and lower bounds of *x*; that is, (*v*_min⁡_, *v*_max⁡_) = (*x*_min⁡_, *x*_max⁡_). If the sum of accelerations would cause the velocity on that dimension *v*(*t* + 1) to exceed *v*_min⁡_ or *v*_max⁡_, then the velocity on that dimension *v*(*t* + 1) will be limited to *v*_min⁡_ or *v*_max⁡_, respectively [6].

#### 4.2.3. ABC Settings

The control parameters of the ABC algorithm are as follows: the maximum number of cycles is equal to the maximum number of generation and the colony size is equal to the population size, that is, 50, as presented in [6]. The percentage of onlooker bees was 50% of the colony, the employed bees were 50% of the colony, and one bee was selected as the scout bee. The increase in the number of scouts encourages the exploration because the increase in onlookers for a food source increases exploitation.

#### 4.2.4. SLO Settings

In SLO, each dimension is divided into two equal parts, and thus, 2^*D*^ patches are generated. In each patch, the initial population of ladybirds is set to 20. The parameter *limit* is 100 and *w* is 1; that is, after 100 cycles of search, if a position in a patch cannot be improved, then it will be abandoned and a new position will be produced in the neighborhood of *g*best. The parameter *c* in (2) and (4) decreases linearly from 10 to 2. The sideward vibration *ε*_1_ is Rand∗10*e* − 4 and *ε*_2_ is Rand∗10*e* − 8.

### 4.3. Results and Discussion

In this paper, all the experiments were repeated 30 times with different random seeds. The best and mean function values of the solutions found using the algorithms for different dimensions were recorded. Tables 1, 2, 3, 4, and 5 present the mean, best, and standard deviations of the function values obtained using SLO, GA, PSO, and ABC with *D* = 5, *D* = 10 and *D* = 30. Figures 4, 5, 6, 7, 8, 9, 10, 11, 12, 13, 14, 15, 16, 17, and 18 show the convergence characteristics in terms of the fitness value of each algorithm for each test function.

According to the best function values obtained using the different algorithms with *D* = 5, the SLO can find the global optimization solution with values close to the theoretical solution and has the same search ability as PSO. Many literatures have pointed out that larger population size and large number of generations increase the likelihood of obtaining a global optimum solution. Thus, the performance of PSO with a swarm size of 50 is better than that with a swarm size of 20. According to the experiments in our paper, SLO with a small population of 20 was able to find the global optimization solution with values close to the theoretical solution. This indicates the proposed SLO algorithm has the ability to find the best solution with a comparatively small population size. Based on the mean results of all experiments, the proposed SLO has better performance than GA for Griewank function, Ackley function, and Schwefel function. However, when dimension is 30, the result of SLO is no better than that of PSO and ABC. Comparing the convergence graphs, SLO converged faster and performed better compared with GA.

From the results, we can see that the SLO does not obtain better result along with the growing dimensions. Considering the No Free Lunch Theorem [41], if we compare two searching algorithms with all possible functions, the performance of any two algorithms will be, on average, the same. As a result, when an algorithm is evaluated, we must look for the kind of problems where its performance is good, in order to characterize the type of problems for which the algorithm is suitable [42]. In this paper, the proposed SLO is suitable for solving optimization problems with lower dimensions.

## 5. Conclusion

This paper investigated the foraging behaviors of seven-spot ladybirds and proposed a novel biologically inspired meta-heuristic algorithm called SLO. The SLO, GA, PSO, and ABC algorithms were tested on five numerical benchmark functions with multimodality to validate the performance of SLO. The simulated results show that SLO has the ability to find the best solution and is suitable for solving optimization problems with lower dimensions. In this paper, the ABC algorithm outperformed all other algorithms, but according to the No Free Lunch Theorem [41], “any elevated performance over one class of problems is offset by performance over another class.” Future studies will focus on improving the SLO.

## Figures and Tables

**Figure 4:**
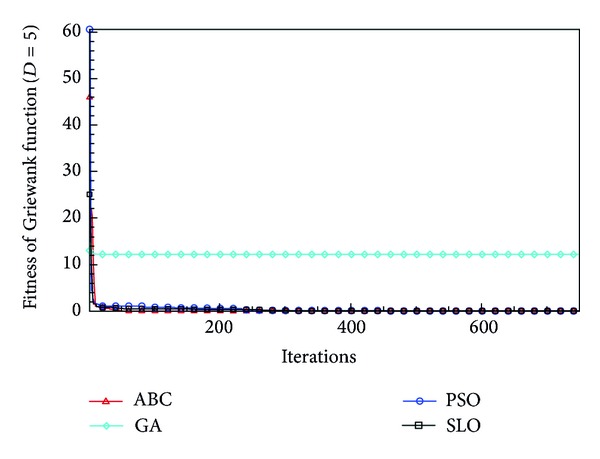
Convergence characteristics of the Griewank function with *D* = 5.

**Figure 5:**
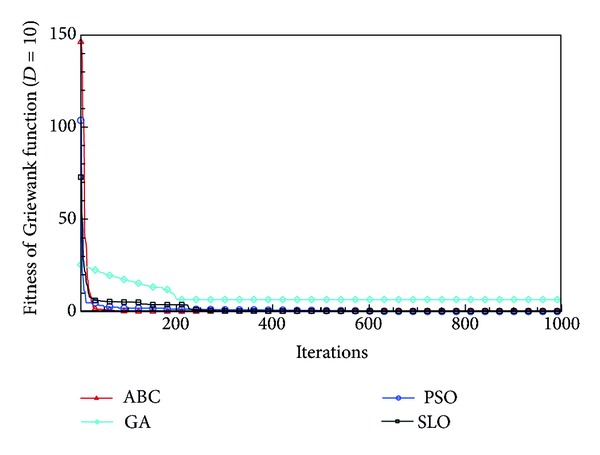
Convergence characteristics of the Griewank function with *D* = 10.

**Figure 6:**
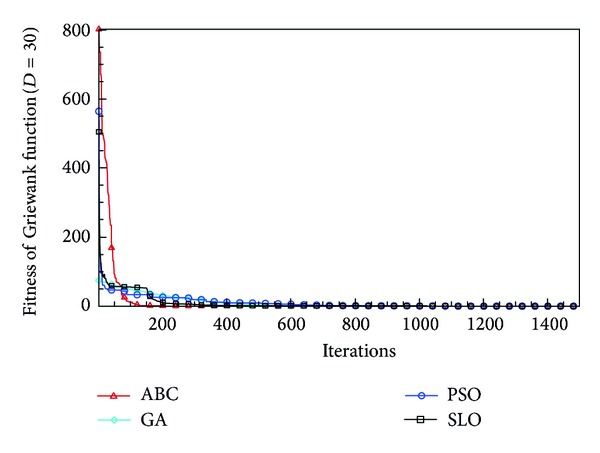
Convergence characteristics of the Griewank function with *D* = 30.

**Figure 7:**
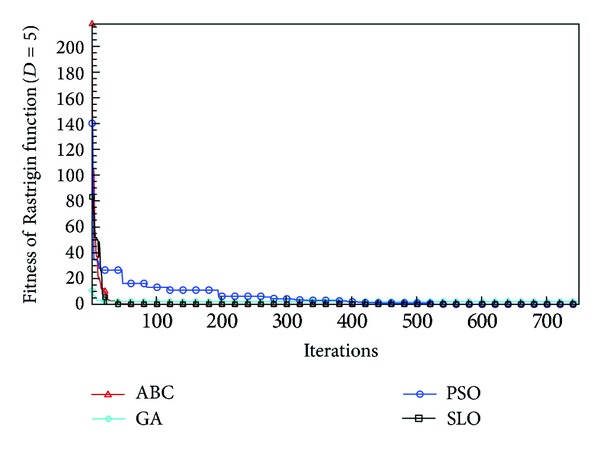
Convergence characteristics of the Rastrigin function with *D* = 5.

**Figure 8:**
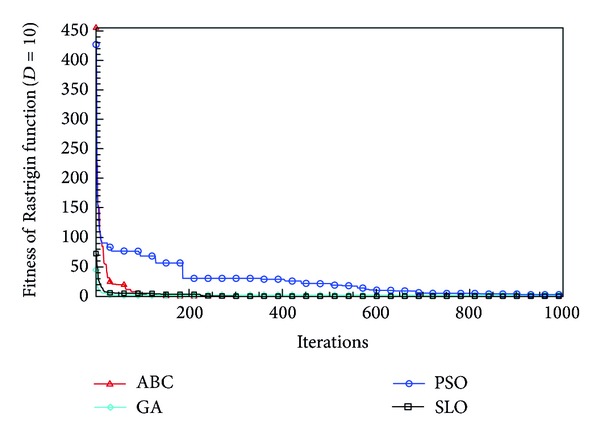
Convergence characteristics of the Rastrigin function with *D* = 10.

**Figure 9:**
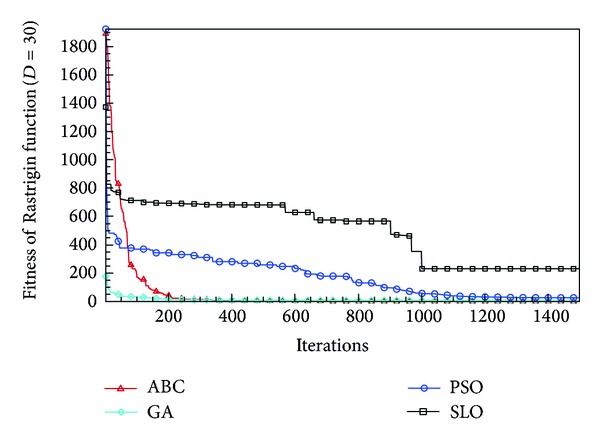
Convergence characteristics of the Rastrigin function with *D* = 30.

**Figure 10:**
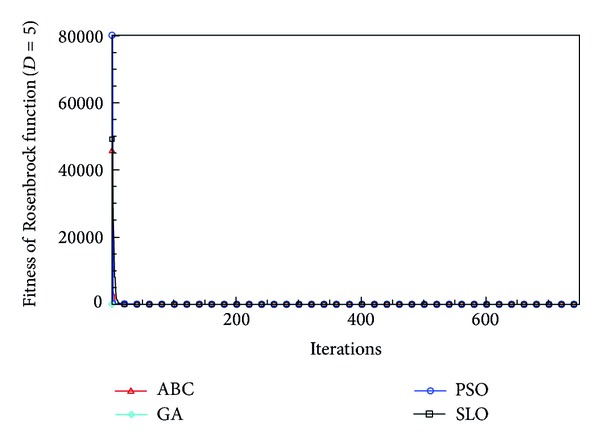
Convergence characteristics of the Rosenbrock function with *D* = 5.

**Figure 11:**
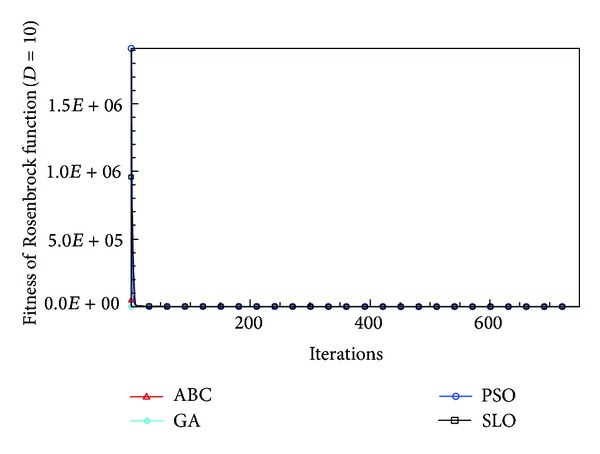
Convergence characteristics of the Rosenbrock function with *D* = 10.

**Figure 12:**
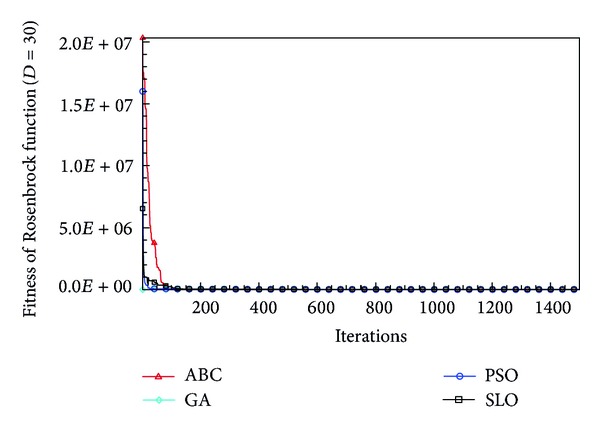
Convergence characteristics of the Rosenbrock function with *D* = 30.

**Figure 13:**
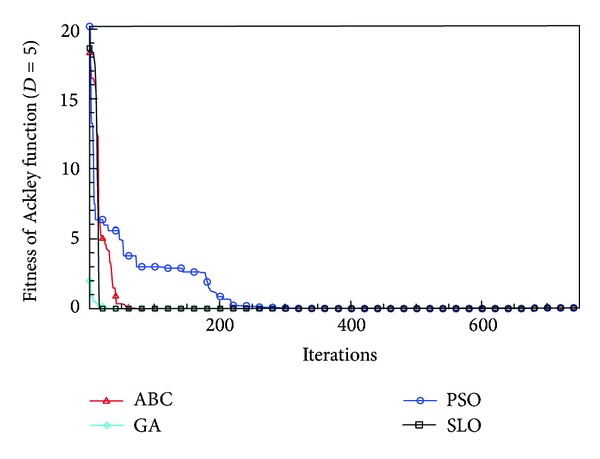
Convergence characteristics of the Ackley function with *D* = 5.

**Figure 14:**
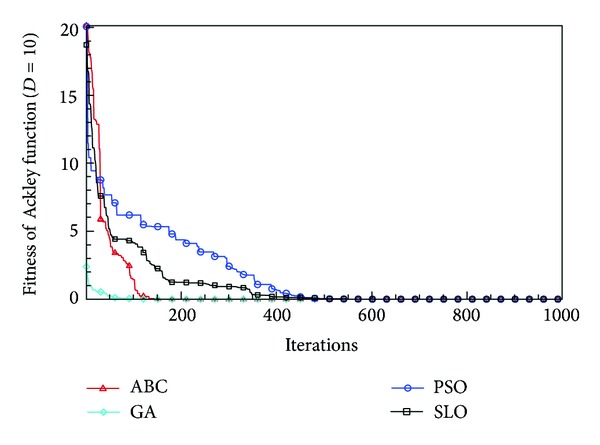
Convergence characteristics of the Ackley function with *D* = 10.

**Figure 15:**
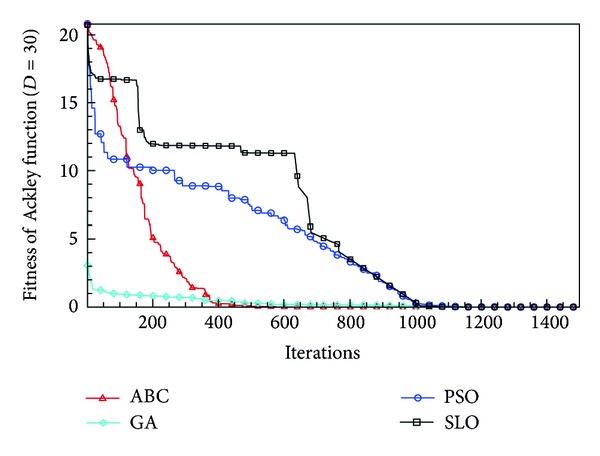
Convergence characteristics of the Ackley function with *D* = 30.

**Figure 16:**
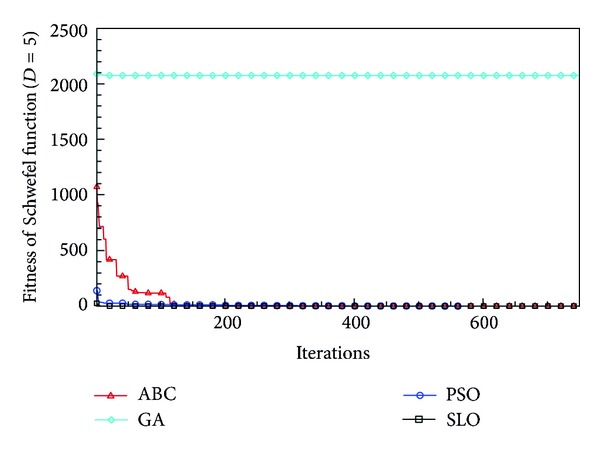
Convergence characteristics of the Schwefel function with *D* = 5.

**Figure 17:**
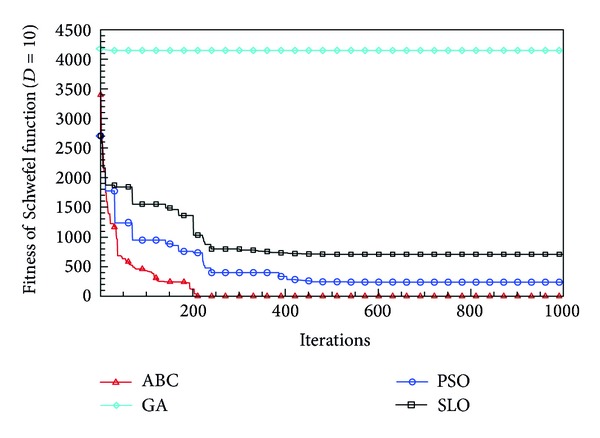
Convergence characteristics of the Schwefel function with *D* = 10.

**Figure 18:**
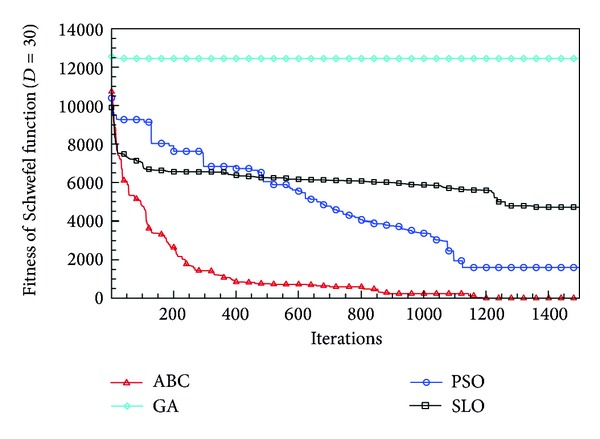
Convergence characteristics of the Schwefel function with *D* = 30.

**Table 1:** Results of the Griewank function.

Algorithm	Dimension	Mean	Best	SD
SLO	5	1.2870*E* − 01	7.4000*E* − 03	9.1200*E* − 02
	10	3.1000*E* − 01	2.4600*E* − 02	5.2890*E* − 01
	30	1.4705*E* + 00	6.2000*E* − 03	1.7894*E* + 00

GA	5	1.2240*E* + 01	1.2240*E* + 01	1.2267*E* − 10
	10	1.4568*E* + 01	6.5624*E* + 00	4.7149*E* + 00
	30	1.4100*E* − 02	1.9119*E* − 10	2.4700*E* − 02

PSO	5	2.3700*E* − 02	7.4000*E* − 03	1.2400*E* − 02
	10	7.6900*E* − 02	2.7000*E* − 02	3.3600*E* − 02
	30	1.3200*E* − 02	1.1102*E* − 15	1.4900*E* − 02

ABC	5	6.8808*E* − 04	0.0000*E* + 00	2.1000*E* − 03
	10	2.8000*E* − 03	1.1102*E* − 16	4.6000*E* − 03
	30	6.3515*E* − 04	0.0000*E* + 00	3.5000*E* − 03

**Table 2:** Results of the Rastrigin function.

Algorithm	Dimension	Mean	Best	SD
SLO	5	0.0000*E* + 00	0.0000*E* + 00	0.0000*E* + 00
	10	2.2781*E* + 01	9.9760*E* − 01	1.4721*E* + 01
	30	3.6123*E* + 02	2.3147*E* + 02	5.9041*E* + 01

GA	5	6.3010*E* − 01	2.0653*E* − 09	6.1180*E* − 01
	10	7.9600*E* − 01	3.3258*E* − 08	8.4260*E* − 01
	30	3.0844*E* + 00	2.5108*E* − 06	2.3711*E* + 00

PSO	5	6.6300*E* − 02	0.0000*E* + 00	2.5240*E* − 01
	10	1.7267*E* + 00	0.0000*E* + 00	1.1662*E* + 00
	30	2.9001*E* + 01	1.7927*E* + 01	8.5603*E* + 00

ABC	5	0.0000*E* + 00	0.0000*E* + 00	0.0000*E* + 00
	10	0.0000*E* + 00	0.0000*E* + 00	0.0000*E* + 00
	30	1.0658*E* − 15	0.0000*E* + 00	4.2397*E* − 15

**Table 3:** Results of the Rosenbrock function.

Algorithm	Dimension	Mean	Best	SD
SLO	5	1.3325*E* + 00	1.6163*E* − 08	8.7800*E* − 01
	10	1.6880*E* + 01	6.6976*E* + 00	2.3646*E* + 01
	30	1.6123*E* + 04	2.5639*E* + 03	9.1648*E* + 03

GA	5	5.0800*E* − 02	9.2000*E* − 03	2.0100*E* − 02
	10	7.0620*E* − 01	1.0590*E* − 01	5.6060*E* − 01
	30	2.3368*E* + 01	7.5000*E* − 02	2.2251*E* + 01

PSO	5	2.2040*E* − 01	3.6080*E* − 04	3.9500*E* − 01
	10	2.6053*E* + 00	3.9800*E* − 02	1.3755*E* + 00
	30	4.1055*E* + 01	1.1177*E* + 01	2.7793*E* + 01

ABC	5	5.1300*E* − 02	4.9000*E* − 03	5.6700*E* − 02
	10	5.2200*E* − 02	1.9000*E* − 03	5.1100*E* − 02
	30	6.0700*E* − 02	3.6401*E* − 04	7.5300*E* − 02

**Table 4:** Results of the Ackley function.

Algorithm	Dimension	Mean	Best	SD
SLO	5	−8.8818*E* − 16	−8.8818*E* − 16	0.0000*E* + 00
	10	9.3400*E* − 02	2.6645*E* − 15	3.6120*E* − 01
	30	9.2670*E* − 01	6.2172*E* − 15	2.3272*E* + 00

GA	5	1.9147*E* − 05	8.7429*E* − 07	1.4740*E* − 05
	10	2.8850*E* − 05	4.1240*E* − 06	1.2182*E* − 05
	30	7.6502*E* − 05	5.3960*E* − 05	1.2228*E* − 05

PSO	5	2.0724*E* − 15	−8.8818*E* − 16	1.3467*E* − 15
	10	3.6119*E* − 15	2.6645*E* − 15	1.5979*E* − 15
	30	1.7064*E* − 08	1.5857*E* − 09	2.1073*E* − 08

ABC	5	2.6645*E* − 15	2.6645*E* − 15	0.0000*E* + 00
	10	6.9278*E* − 15	2.6645*E* − 15	2.1681*E* − 15
	30	4.4154*E* − 13	1.6964*E* − 13	3.0035*E* − 13

**Table 5:** Results of the Schwefel function.

Algorithm	Dimension	Mean	Best	SD
SLO	5	3.2505*E* + 02	6.3638*E* − 05	1.7285*E* + 02
	10	1.2493*E* + 03	7.5042*E* + 02	2.7913*E* + 02
	30	5.8700*E* + 03	4.7145*E* + 03	7.6059*E* + 02

GA	5	2.0752*E* + 03	2.0752*E* + 03	5.2257*E* − 11
	10	4.1504*E* + 03	4.1504*E* + 03	9.4875*E* − 11
	30	1.2451*E* + 04	1.2451*E* + 04	7.3221*E* − 10

PSO	5	2.9610*E* + 02	6.3638*E* − 05	1.3104*E* + 02
	10	5.6719*E* + 02	2.3688*E* + 02	1.6709*E* + 02
	30	2.8564*E* + 03	1.5989*E* + 03	4.0442*E* + 02

ABC	5	6.3638*E* − 05	6.3638*E* − 05	1.9653*E* − 14
	10	1.2728*E* − 04	1.2728*E* − 04	5.2425*E* − 14
	30	3.1000*E* − 03	3.8183*E* − 04	1.4200*E* − 02
